# Identification and validation of the N6-methyladenosine RNA methylation regulator ZC3H13 as a novel prognostic marker and potential target for hepatocellular carcinoma

**DOI:** 10.7150/ijms.69645

**Published:** 2022-03-21

**Authors:** Shuang Wu, Guifang He, Shihai Liu, Yongxian Cao, Chao Geng, Huazheng Pan

**Affiliations:** 1 Department of Clinical Laboratory, The Affiliated Hospital of Qingdao University, Qingdao, Shandong 266003, People's Republic of China.; 2 Department of Medicine, Qingdao University, Qingdao, Shandong 266071, People's Republic of China.; 3 Medical Animal Laboratory, The Affiliated Hospital of Qingdao University, Qingdao, Shandong 266003, People's Republic of China.

**Keywords:** ZC3H13, Hepatocellular Carcinoma, Prognostic value, M6A-related genes, Bioinformatics analyses

## Abstract

N6-methyladenosine (m6A) RNA methylation has been implicated in various malignancies. This study aimed to identify prognostic signature based on m6A methylation regulators for hepatocellular carcinoma (HCC) and provide candidate targets for HCC treatment. In this study, the expression levels, prognostic values, correlation with tumor grades and genetic variations of m6A-related genes in HCC were evaluated using bioinformatics analyses. Interestingly, the results show that methyltransferase zinc finger CCCH-type containing 13 (ZC3H13) was expressed at a significantly low level in HCC. Survival outcome analysis suggested that significant correlations existed between ZC3H13 downregulation and poor overall survival (OS) and poor recurrence-free survival (RFS) in HCC patients. Therefore, ZC3H13 was chosen for further experimental validation. The expression of ZC3H13 in HCC cell lines was investigated by western blotting. Knockdown of ZC3H13 significantly enhanced the migration and invasion of HCC cells, as demonstrated by wound healing and transwell assays. Moreover, upregulating ZC3H13 repressed the growth of xenograft tumors *in vivo*. Functional and pathway enrichment analyses indicated that ZC3H13 might be involved in transcriptional dysregulation or the JAK-STAT signaling pathway in cancer. Additionally, ZC3H13 expression was significantly correlated with lymphocytes and immunomodulators. Therefore, ZC3H13 is a promising candidate as a novel biomarker and therapeutic target for HCC.

## Introduction

Primary liver cancer is the sixth most commonly diagnosed cancer and the third leading cause of cancer death worldwide in 2020, with approximately 906,000 new cases and 830,000 deaths. Primary liver cancer includes hepatocellular carcinoma (HCC) (comprising 75%-85% of cases) and intrahepatic cholangiocarcinoma (10%-15%), as well as other rare types 1. Early diagnosis and multidisciplinary treatment are critical to improve HCC prognosis 2. Early diagnosis largely depends on monitoring high-risk patients as well as advances in diagnostic techniques. The rapid progression of imaging techniques (such as diffusion-weighted magnetic resonance imaging) has provided us powerful tools for monitoring and diagnosing chronic hepatitis and HCC 3. Multidisciplinary treatments offer the best treatment efficacy based on currently available therapies 2-4. However, treatment outcomes for HCC are far from satisfactory, mainly due to two major barriers: low rates of receiving potentially curable treatments and high rates of recurrence or metastasis after treatment 5. In order to further improve the prognosis of HCC, we need to explore the detailed molecular mechanisms of oncogenesis and cancer progression.

N6-methyladenosine (m6A), the methylation modification at the sixth N atom of adenine, is the most common post-transcriptional modification on mRNA, mediating > 60% RNA methylation 6. Abnormal m6A methylation levels are closely related to stem cell differentiation, immune response, embryonic development, and microRNA (miRNA) editing; they also play an essential role in the progression of various cancers 7. The epigenetic modification of m6A methylation affects tumor development by modulating the mRNA expression levels of related oncogenes or suppressor genes. The m6A methylation levels in tumors mainly depend on the expression of m6A methylation regulators. The m6A methylation is highly correlated with the expression of intracellular methyltransferases (“writers”) and demethylases (“erasers”), whereas binding proteins (“readers”) bind to m6A methylation sites in performing a series of biological functions 8, 9. The “Writers,” which included methyltransferase like 16 (METTL16), Wilms tumor 1 associated protein (WTAP), zinc finger CCCH-type containing 13 (ZC3H13), KIAA1429 and RNA-binding motif protein 15 (RBM15), promote m6A RNA methylation 10, 11. The “Erasers,” comprising fat mass- and obesity-associated protein (FTO), alkb homolog 5 (ALKBH5), remove m6A methylation groups from RNA 12. The “Readers,” which include YTH domain-containing 1 (YTHDC1), YTH N6-methyl-adenosine RNA-binding protein 1 (YTHDF1), YTHDF2, YTHDF3, and heterogeneous nuclear ribonucleoprotein C (HNRNPC), play a specific role by binding to the m6A methylation site 13. The aberrant expression of m6A regulators plays a vital regulatory role in tumor progression, prognosis, and radio-resistance 12.

Some studies indicated that m6A regulators were related to poor prognosis of HCC patients and promoted the malignant phenotypes of HCC cells. For example, KIAA1429 was shown to facilitate cell proliferation and invasion of HCC cells through m6A modification of ID2 mRNA and GATA3 pre-mRNA 14. WTAP-mediated m6A modification contributed to the aggressiveness of HCC cells via posttranscriptional suppression of ETS proto-oncogene 1 15. Interestingly, YTHDF2 was described as an HCC suppressor by repressing cell proliferation via m6A modification of epidermal growth factor receptor (EGFR). YTHDF2 also inhibited vascular reconstruction and metastasis via regulating interleukin 11 and serpin family E member 2 16. Bian et al. found that YTHDF1 was further identified as an oncogenic gene for HCC by facilitating AKT/GSK3β/β-catenin signaling 17. Moreover, Lin et al. revealed a critical function for METTL3-mediated m6A modification in the hypoxic tumor microenvironment and identifies FOXO3 as an important target of m6A modification in the resistance of HCC to sorafenib therapy 18. Despite these studies, the clinical significance of these m6A regulators in HCC remains unclear and poorly explored. In this study, we aimed to investigate the expression characteristics and clinicopathological value of the m6A RNA regulators comprehensively in order to identify robust risk signatures for HCC prognosis and potential targets for HCC treatment.

Here, we searched for mRNA expression levels of m6A-related genes between hepatocellular carcinoma and normal liver tissues using the Oncomine and Gene Expression Profiling Interactive Analysis (GEPIA) databases, analyzed the prognostic value of each m6A family member in liver cancer using the Kaplan-Meier plotter database. We further analyzed ZC3H13, which was significantly correlated with the prognosis of patients with hepatocellular carcinoma. Furthermore, ZC3H13 was further identified as tumor suppressor gene for HCC by functional assays *in vitro* and *in vivo*. Then, ZC3H13 was subjected to gene set enrichment analysis using The Cancer Genome Atlas (TCGA) database to explore its biological functions and relevant pathways. Correlation between ZC3H13 and the immune system were analyzed using the TISIDB database. Our study was aimed at exploring the clinical significance of ZC3H13 and providing a theoretical basis for the early diagnosis, prognostic judgments, and targeted therapy of hepatocellular carcinoma.

## Materials and Methods

### TCGA and cBioPortal analyses

The open-source cBioPortal database (http://www.cbioportal.org) contains data retrieved from the TCGA database for interactively exploring cancer genomic datasets 19. It contains a wide variety of data, including DNA Keywords: ZC3H13, hepatocellular carcinoma, prognostic value, m6A-related genes. Bioinformatics analyses include copy numbers, DNA methylation, mRNA and microRNA expression levels, and nonsynonymous mutations. In this study, the database was used to evaluate correlations between m6A-related genes, after which these data were normalized, and then functional and pathway enrichment analysis was performed for ZC3H13 using the R package ggplot2. A p value < 0.05 was considered statistically significant.

### Oncomine analysis

The Oncomine database (http://www.oncomine.org) is an online microarray database that includes 715 datasets, as well as 86,733 cancer and normal tissue samples 20. In this study, the Oncomine database was employed to analyze the mRNA expression levels of m6A-related genes in HCC and liver tissue. The search was carried out based on the following criteria: (a) type of analysis: cancer versus normal tissues; (b) type of data: mRNA; (c) thresholds: fold change = 2 and p value = 0.01.

### GEPIA dataset analysis

GEPIA (http://gepia.cancer-pku.cn/) is a database of data retrieved from the UCSC Xena server, which includes 9736 tumor samples and 8587 normal samples. The database can be used to analyze differential gene expression levels in tumor tissues and paracancerous tissues, as well as patient survival and prognosis 21. In this study, we validated the differential mRNA expression levels of m6A-related genes in cancerous and paracancerous tissues of HCC using the database. P < 0.05 indicated statistically significant differences.

### Kaplan-Meier plotter analysis

As a tool for assessing biomarkers, Kaplan-Meier plotter (http://kmplot.com) can be used to assess the functions of 54,675 genes and 10,188 tumor tissue samples, including breast cancer, liver cancer, lung cancer and gastric cancer 22. In this study, we analyzed the prognostic value of m6A-related genes in HCC using Kaplan-Meier plotter. The prognostic values of the high- and low-expression groups were evaluated according to the hazard ratio (HR), 95% confidence interval (CI), and log-rank p values. P < 0.05 indicated statistically significant differences.

### GeneMANIA analysis

GeneMANIA (http://www.genemania.org) provides a flexible web interface for deriving hypotheses based on gene functions. GeneMANIA generates a list of genes with similar functions to the query gene and constructs an interactive functional-association network to illustrate relationships between genes and datasets 23. In this study, a database was adopted to construct a gene-gene interaction network for m6A-related genes in terms of physical interactions, co-expression, predictions, colocalization, and genetic interaction, as well as to evaluate their functions.

### Functional and pathway enrichment analysis

We identified genes co-expressed with ZC3H13 using the cBioPortal database. A Spearman's correlation coefficient exceeding 0.50 indicated a good correlation between ZC3H13 and co-expressed genes. Metascape (https://metascape.org/) is a free and well-maintained online bioinformatics database for Gene Ontology (GO) and Kyoto Encyclopedia of Genes and Genomes (KEGG) enrichment analysis 24. In this study, we used Metascape for functional annotation and pathway enrichment analysis of ZC3H13 and co-expressed genes significantly associated with ZC3H13. Additionally, data from the cBioPortal database were normalized, and differential expression analysis was performed for ZC3H13 using the R package ggplot2 and clusterProfiler. Only terms with a p value < 0.05, minimum count > 3, and enrichment factor > 1.5 were considered significant.

### TISIDB analysis

The TISIDB database (http://cis.hku.hk/TISIDB) integrates 988 reported immune-related anti-tumor genes, high-throughput screening techniques, molecular profiling, and paracancerous multiomics data, as well as various resources for immunological data retrieved from seven public databases. The database enables analyses of correlations for selected genes with lymphocytes, immunomodulators, and chemokines 25. In this study, we employed the TISIDB database to analyze the relationship between the expression levels of m6A-related genes and tumor grades in HCC and to investigate correlations between ZC3H13 expression and lymphocytes and immunomodulators.

### LinkedOmics

LinkedOmics is a bioinformatics web portal designed for accessing, analyzing, and comparing cancer multiomics data of various types of cancer 26. We submitted ZC3H13 to TCGA datasets of 371 liver hepatocellular carcinoma (LIHC) patients and analyzed ZC3H13-associated genes using the Pearson correlation test. In the “Link-Interpreter” module, gene set enrichment analysis (GSEA) was conducted to explore the enrichment function of ZC3H13 and neighboring genes with 3 as the minimum number of genes and 0.05 as the p value. Enrichment analysis involved GO and KEGG pathway analysis.

### GSCALite

As a bioinformatics platform for gene set cancer analysis, GSCALite offers several types of analyses, including methylation analysis, cancer-related pathway analysis, miRNA network analysis, etc. 27. In the current study, GSCALite was used to analyze the CNV profile of the m6A-related genes in LIHC. All analyses were performed using the LIHC TCGA dataset.

### Nomogram

A nomogram was constructed based on the risk factors affecting prognosis using a Cox proportional hazards regression model. Risk factors included in clinically recognized TNM staging and candidate factor ZC3H13. And we choose to predict 3 survival times, namely 1, 3, and 5 years. We utilized R software (version 3.6.3) to conduct the statistical analysis. The “rms” package was downloaded from The Comprehensive R Archive Network (tsinghua.edu.cn).

### Cell Culture and Transfection

The cell lines used in this study were purchased from American Type Culture Collection. These cell lines include LX-2 (human astrocyte cell line), HepG2, Hep3B, SNU-398 and Huh7 cells. All cancer cells were maintained in high-glucose Dulbecco's modified Eagle medium (DMEM; Thermo Scientific) supplemented with 10% fetal bovine serum (FBS, Gibco), 0.1 mmol/L MEM nonessential amino acids (NEAA; Invitrogen), and 1% L-glutamine (Invitrogen). All cell lines were cultured in 5% CO_2_ at 37°C in incubators at 100% humidity.

A GV141 vector containing ZC3H13 was purchased from GeneChem (GeneChem, Shanghai, China). The GV141-ZC3H13 or GV141 control vector was transfected into target HCC cells using Lipofectamine 3000 (Thermo) transfection reagent according to the manufacturer's instructions.

### Western Blotting

Cells were washed with PBS twice before radioimmunoprecipitation assay (RIPA) lysis solution (100 µL) was added, followed by 5× loading buffer; then, the solution was fully mixed and boiled for 5 minutes at 95°C before being stored at -20°C. Protein concentration was determined using the Protein Concentration Assay Kit (Keygene, Nanjing, China). For western blotting assays 30 µg of protein sample was separated using sodium dodecyl sulfate-polyacrylamide gel electrophoresis, transferred to polyvinylidene difluoride membranes, and sealed with 5% skimmed milk powder for 2 hours. After adding the primary antibody, the membranes were shaken overnight at 4°C. The membranes were washed three times with Tris-buffered saline-Tween (TBST), incubated with secondary antibodies for 2 hours at room temperature, washed three times with TBST, and then developed and exposed using enhanced chemiluminescence.

### Migration and Invasion Assays

The migration and invasion of cancer cells were analyzed using the transwell system (Corning, NY, USA). Briefly, bladder cancer cells (1 × 10^5^ cells/ml) were seeded in the upper chamber of transwell plates containing serum-free DMEM, and the lower chambers were filled with DMEM with 10% FBS. Cells that migrated to the lower chambers after normal culture for 48 h were stained with 1% crystal violet solution for 12 min and photographed under microscopy to assess migration. To evaluate the invasive capacity, cancer cells were seeded in transwell plates pre-coated with Matrigel matrix (Corning, NY, USA). The rest of the assay was carried out following the same experimental procedures.

### Xenograft Tumor Assay

Six-week-old male BALB/c nude mice were purchased from Beijing Vital River Laboratory Animal Technology Co., Ltd. and maintained in a pathogen-free experimental facility with free access to food and drinking water. The Experimental Animal Ethics Committee of the Affiliated Hospital of Qingdao University approved all experiments in advance (AHQU-MALF20210210). The effect of both upregulation and knockdown of ZC3H13 were evaluated. Mice were randomly divided into four groups of six. First, the tumorigenesis of HCC cancer cells was assessed through the cancer cell line-based xenograft (CDX). The expression of ZC3H13 was modified in HCC cancer cells by transfection, and the cultured cells were introduced into the rear flank of the nude mouse by subcutaneous injection (2 × 10^6^ cells/mouse). The size and weights of tumors formed in nude mice were measured 24 days after cell injection.

### Statistical analysis

The statistical analysis in this study was automatically performed by the online database mentioned above. A p value < 0.05 or log rank p value < 0.05 was considered statistically significant.

## Results

### Bioinformatics analyses revealed differential mRNA expression levels of m6A-related genes in HCC

**Figure 1:**
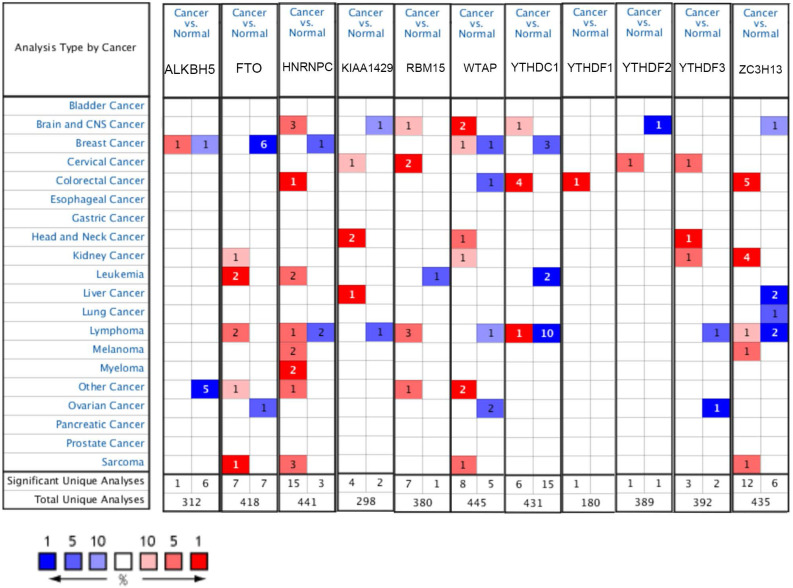
mRNA expression levels of m6A-related genes in various types of cancer (Oncomine). The threshold was set with following parameters: fold change = 2 and p value = 0.01. The cell number represents the number of datasets that meet the thresholds. The color intensity (red or blue) is directly proportional to the significance level of upregulation or downregulation, respectively.

**Figure 2:**
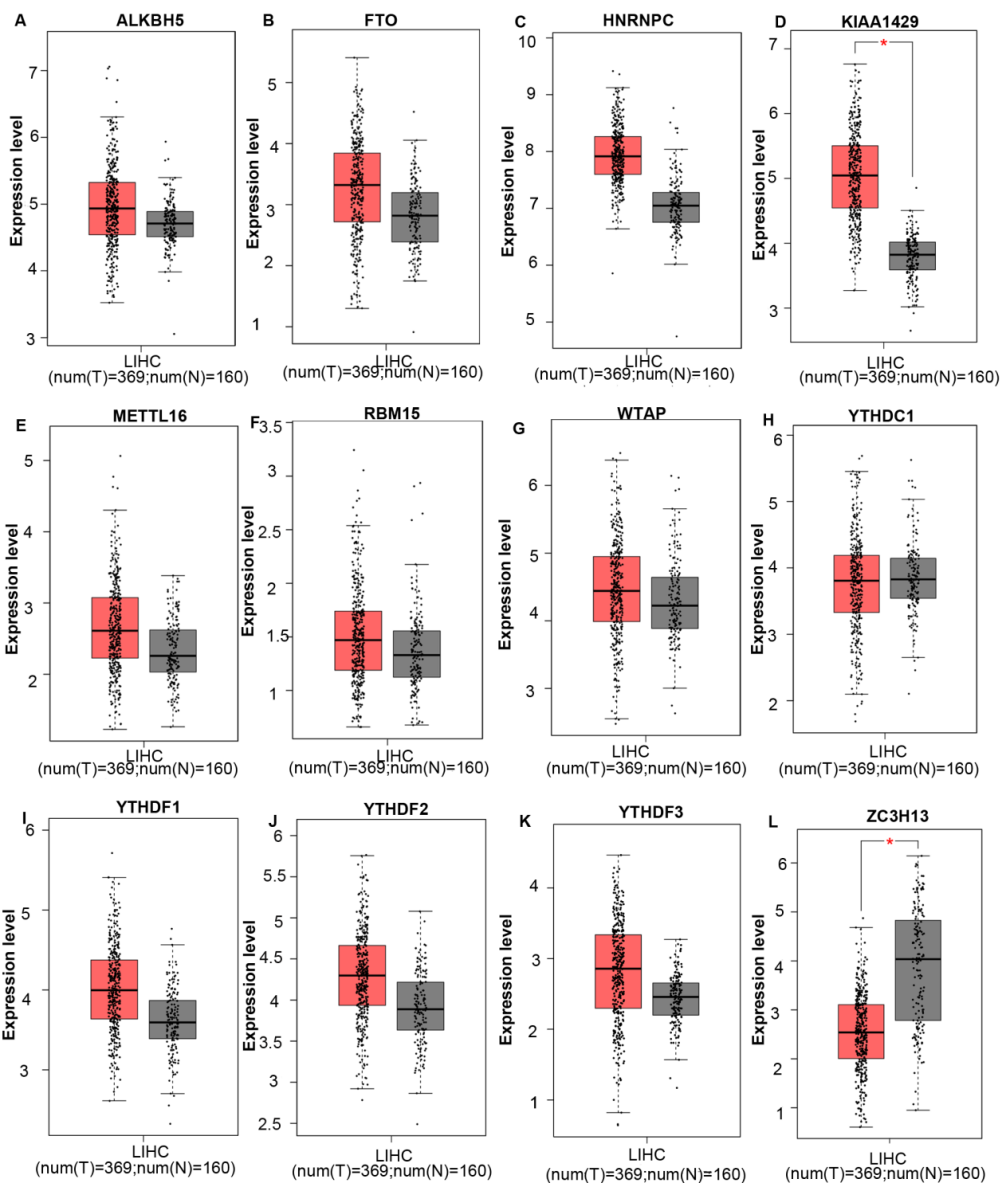
mRNA expression levels of m6A-related genes in liver cancer and normal liver tissues (GEPIA). **(A-L)** mRNA expression levels of each member of the m6A-related genes in liver cancer and normal liver tissues. Box plots show mRNA expression of liver cancer (red plot) and the corresponding normal tissues (gray plot). Axis units are Log2 (TPM + 1). *p < 0.01.

The mRNA expression levels of m6A-related genes in various cancer vs normal were analyzed using the Oncomine database (Figure 1). The expression of ZC3H13 is up-regulated in patients with colorectal cancer, kidney cancer, melanoma and sarcoma. However, the expression of ZC3H13 is down-regulated in patients with brain and CNS cancer, liver cancer and lung cancer. In the Roessler dataset, by comparing the mRNA expression levels of 22 HCC and 21 normal liver tissues (Table 1), it was found that ZC3H13 was significantly down-regulated in malignant tumor tissues (p = 1.54E-11, fold change = -3.105). We found that KIAA1429 was lowly expressed in patients with brain and CNS cancer and lymphoma. However, high expression of KIAA1429 in cervical cancer, brain and neck cancer and liver cancer patients. Wurmbach dataset indicated that KIAA1429 was upregulated based on 35 HCC cases (p = 1.17E-09, fold change = 2.117). These results are generally consistent with those obtained using the GEPIA database. In detail, we analyzed the differential mRNA expression levels of m6A-related genes between HCC and normal liver tissues using the GEPIA database (Figure 2), which indicated that KIAA1429 expression levels were significantly upregulated in HCC (Figure 2D). In contrast, ZC3H13 expression was significantly downregulated (Figure 2L). Both databases showed that KIAA1429 mRNA expression levels were upregulated in liver cancer compared with normal tissues, while ZC3H13 mRNA expression levels were downregulated.

We then assessed the correlation between the expression of m6A-related genes and the pathological stage of liver hepatocellular carcinoma patients and found a significant correlation between the expression of HNRNPC (Spearman: ρ = 0.207, p = 6.58e-05), KIAA1429 (Spearman: ρ = 0.181, p = 0.000507), YTHDF1 (Spearman: ρ = 0.175, p = 0.000795) and pathological grade. As LIHC progressed, the expression of ZC3H13 (Spearman: ρ = -0.186, p = 0.000347), FTO (Spearman: ρ = -0.15, p = 0.00397) and ALKBH5 (Spearman: ρ = -0.129, p = 0.0135) decreased. In addition, the expression levels of METTL16, RBM15, WTAP, YTHDC1, YTHDF2, and YTHDF3 did not change significantly with grade (Figure S1). These data suggested that these m6A-related genes played significant roles in the development of LIHC.

When we combined the results of three databases, we found that the ZC3H13 mRNA expression level was downregulated in liver cancer, and the expression level decreased significantly as the tumor grade increased. In addition, the KIAA1429 mRNA expression level was upregulated in liver cancer, and the expression levels increased significantly with the tumor grade of HCC.

### Genetic alteration analysis of m6A-related genes in HCC and construction of a gene-gene interaction network

**Figure 3:**
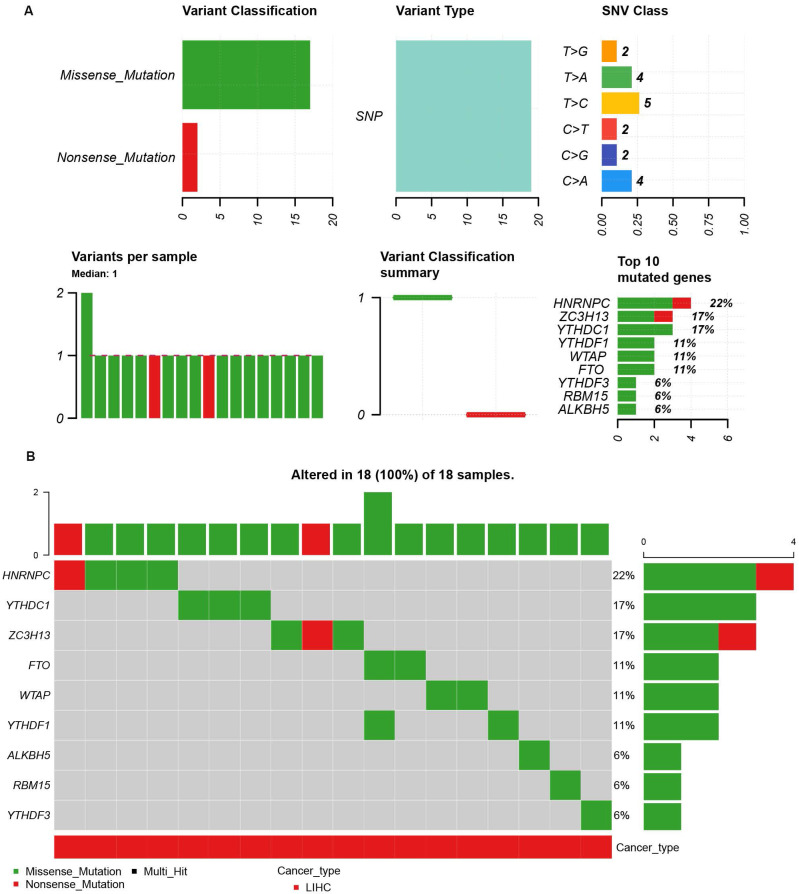
The single nucleotide variation (SNV) analysis of m6A-related genes in LIHC (GSCALite). **(A)** Summary plot displays SNV frequency and variant types of m6A-related genes in LIHC, and genetic alteration of m6A-related genes comprises missense mutation and nonsense mutation.** (B)** Waterfall plot shows the mutation distribution of m6A-related genes in LIHC and HNRNPC (22%), YTHDC1 (17%), and ZC3H13 (17%) were the top three frequently mutated genes among all the numbers of m6A-related genes.

Because of the importance of the m6A family members in LIHC, we investigated whether m6A family members were genetically altered in cancer cells. As shown in Figure 3A, the m6A-related genes showed single nucleotide polymorphisms (SNPs). The altered forms and their frequency are shown in Figure 3B. Among all members of the m6A family, HNRNPC (22%), YTHDC1 (17%), and ZC3H13 (17%) were the top three most frequently mutated genes (Figure 3B). The most common genetic alterations were missense mutations (Figure 3B). A gene-gene interaction network for the 11 m6A-related genes was constructed, and their functions were analyzed using the GeneMANIA database. The 11 central nodes representing m6A family members were surrounded by 20 nodes representing genes that greatly correlated with the family in terms of physical interactions, co-expression, predictions, co-localization, and genetic interactions. A series of genes associated with the m6A family are shown in Figure S2 according to their association. The top five genes displaying the greatest correlations with the m6A family included ALKBH7 (alkB homolog 7), YTHDC2 (YTH domain containing 2), CBLL1 (Cbl proto-oncogene like 1), ALKBH3 (alkB homolog 3, alpha-ketoglutarate dependent dioxygenase), and ALKBH6 (alkB homolog 6), among which CBLL1 was correlated with WTAP and KIAA1429 in terms of physical interactions and co-localized with HEATR6. ALKBH7 was correlated with ALKBH5 in terms of shared protein domains. ZC3H13 was correlated with WTAP and KIAA1429 in terms of physical interactions and predicted. ZC3H13 was connected with YTHDC1 and YTHDF3 in terms of co-expression.

### ZC3H13 showed the greatest prognostic value in patients with HCC

**Figure 4:**
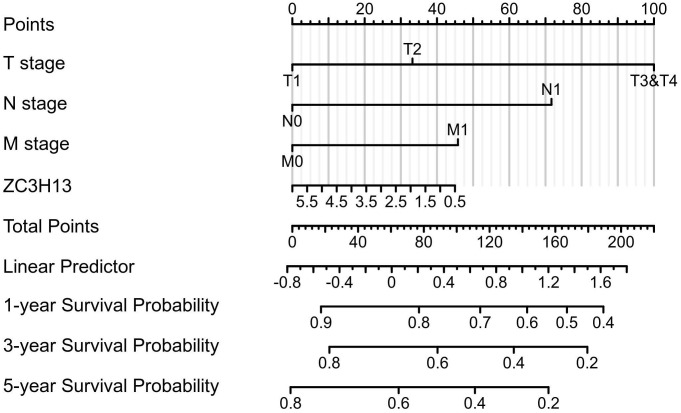
Nomogram integrating the ZC3H13 and clinical characteristics.

To validate the prognostic value of the m6A family members in LIHC, the Kaplan-Meier Plotter platform was used to determine the association between the survival of LIHC patients and the expression of m6A-related genes. The analysis revealed that the mRNA expression levels of ALKBH5, WTAP, YTHDF1, YTHDF2, YTHDF3 and ZC3H13 were correlated with patient prognosis. We observed that high ZC3H13 (Figure S3) mRNA expression was associated with better overall survival (OS) (p = 7.9e-05) and recurrence-free survival (RFS) (p = 0.026). The expression level of KIAA1429 was correlated with OS (p = 0.022) in HCC patients, but RFS (p = 0.059) was not. Therefore, ZC3H13 may act as a potential prognostic biomarker in LIHC. To better predict the 5-y survival rate of HCC patients, we constructed a nomogram that integrated the prognostic model and clinical determinants, including TNM stage. As shown in Figure 4, with each item assigned a score based on the actual condition, patients could get a total score for predicting their survival rate within 5-y.

### The contribution of ZC3H13 to the aggressive behavior of HCC cells

**Figure 5:**
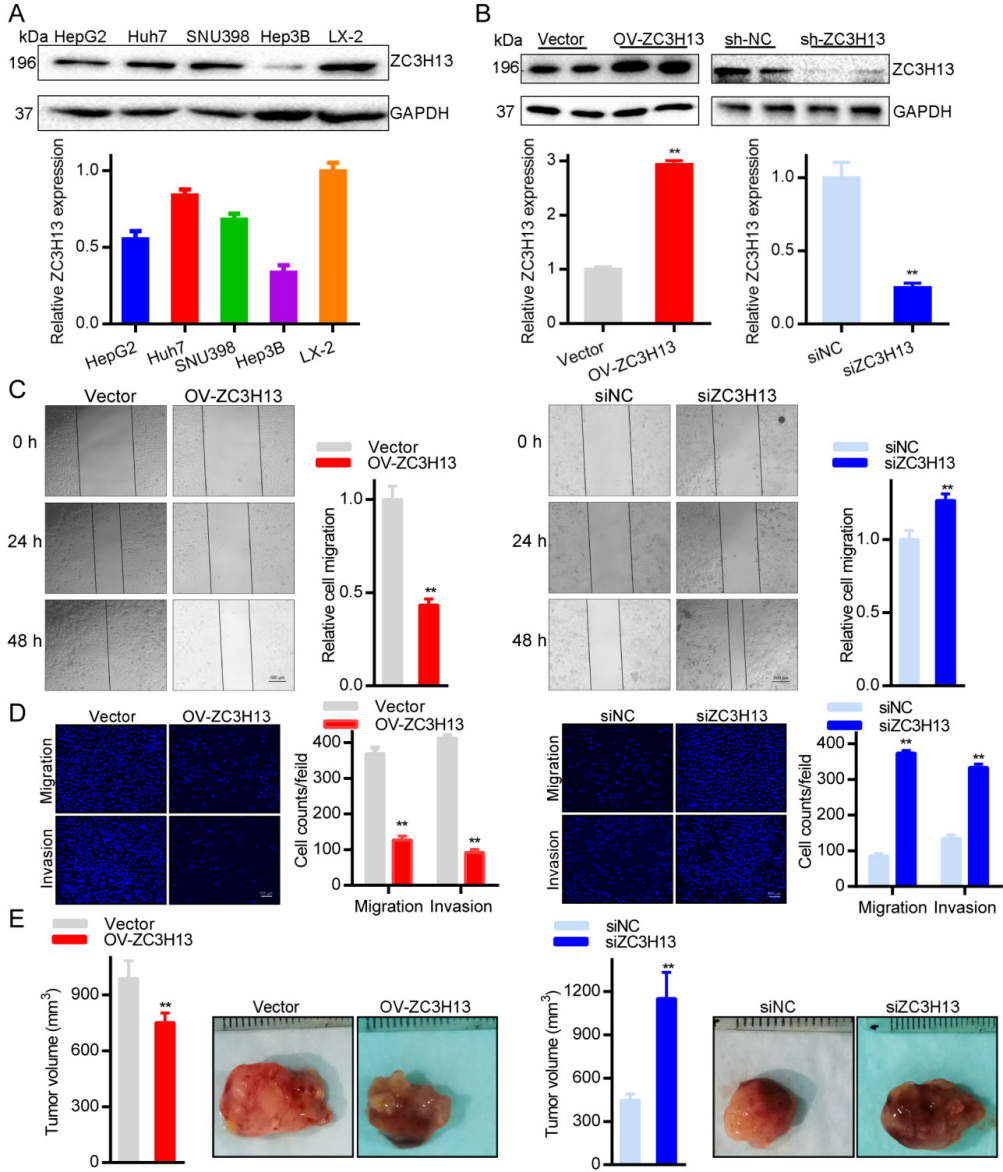
Upregulating ZC3H13 inhibited the aggressive behaviors of HCC cells. **(A)** The expression of ZC3H13 in HCC cell lines was investigated by western blotting. **(B)** Western blotting analysis of the expression of ZC3H13 in HCC cells transiently transfected with siRNA or with the overexpression construct of ZC3H13.** (C)** Migration of Huh7 and Hep3B cells were evaluated by wound-healing assay. **(D)** Invasion of Huh7 and Hep3B cells were evaluated by transwell assay.** (E)** The effects of ZC3H13 on tumor growth were evaluated in a xenograft tumor model in nude mice (n=6). *p < 0.05, **p < 0.01.

To further explore the roles of ZC3H13 in HCC, the current study conducted functional assays *in vitro* and *in vivo*. First, we examined the protein and mRNA expression of ZC3H13 in LX-2 and four (HepG2, Huh7, SNU398, Hep3B) HCC cell lines, in which LX-2 represents normal hepatocytes (Figure 5A). Interestingly, Huh7 cells expressed the highest ZC3H13 level, and Hep3B cells expressed the lowest ZC3H13 level. Therefore, ZC3H13 was upregulated in Hep3B cells and downregulated in Huh7 cells. Western blotting was used to analyze the expression of ZC3H13 in HCC cells transiently transfected with knockdown (Huh7) or overexpression (Hep3B) vectors (Figure 5B). Following upregulation of ZC3H13, migration (p < 0.01) and invasion (p < 0.01) were significantly inhibited, and vice versa (Figures 5C, D). Furthermore, knockdown of ZC3H13 significantly increased the volume of xenograft tumors (p < 0.01, Figure 5E). These pieces of evidence suggest the correlation of ZC3H13 with aggressive phenotypes of HCC cells.

### Functional and pathway enrichment analyses of ZC3H13

**Figure 6:**
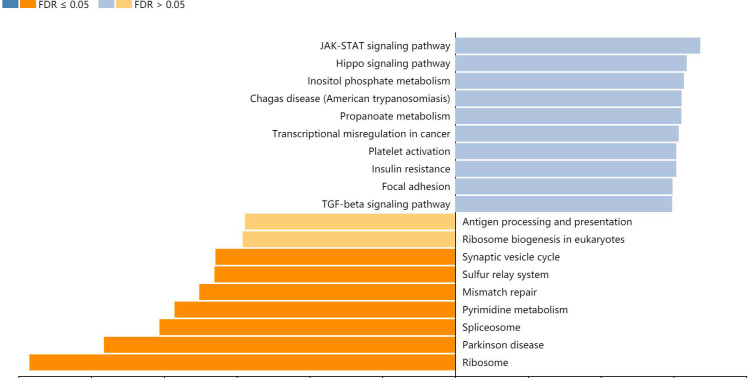
KEGG analysis of ZC3H13 in HCC (LinkedOmics).

A total of 357 genes co-expressed with ZC3H13 with an average Spearman's correlation coefficient of 0.5 were identified from the cBioPortal database based on the selection criteria (Table S1). In this study, the genes co-expressed with ZC3H13 were subjected to functional and pathway enrichment analyses using the R package ggplot2. Bubble plots represent the top 20 enriched biological functions and pathways (based on p value). The functional analysis results showed that the following biological processes were enriched in the genes co-expressed with ZC3H13: transcriptional misregulation in cancer (Figure S4). We also used Metascape to perform functional annotation and pathway enrichment analysis of ZC3H13 and its adjacent genes. The first 20 items of GO enrichment were mainly distributed in the biological process (11 items), Reactome Gene Sets (7 items), CORUM (1 item) and Canonical Pathways (1 item) categories (Figure S4). Five of the first seven projects were in the biological process, which were cellular protein catabolic process; translational elongation; mRNA processing; protein modification by small protein removal; and viral transcription, and the other two were macroautophagy and negative regulation of cell cycle. Canonical pathways referred to ST JNK MAPK pathway. Signaling by TGFB family members included Reactome Gene Sets. The first 100 items of GO enrichment were mainly distributed in the biological process, Reactome Gene Sets, CORUM and Canonical Pathways (Figure S5). Among the signaling pathways showing the greatest correlation with genes co-expressed with ZC3H13 was the KEGG pathway hsa05202 (transcriptional misregulation in cancer). In addition, we analyzed ZC3H13-associated genes using the Pearson correlation test in LinkedOmics (Figure 6). It is noteworthy that the KEGG results of transcriptional misregulation in cancer and the JAK-STAT signaling pathway are common across multiple databases as the function of the genes associated with ZC3H13.

### Regulation of immune molecules by ZC3H13

**Figure 7:**
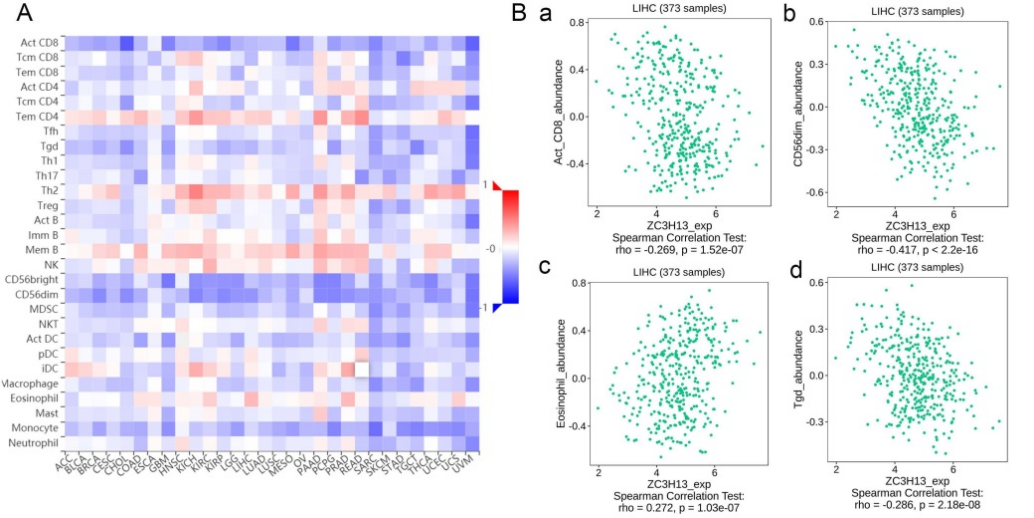
Spearman's correlation of ZC3H13 with lymphocytes and immunomodulators (TISIDB) in HCC. **(A)** Relations between the abundance of TILs and ZC3H13 expression. **(B)** Top 4 TILs displaying the greatest Spearman's correlation with ZC3H13 expression. Red and blue cells indicate positive and negative correlations, respectively. The color intensity is directly proportional to the strength of the correlations. TILs tumor-infiltrating lymphocytes.

Spearman's correlations between ZC3H13 expression and lymphocytes and immunomodulators were analyzed using the TISIDB database (Figure 7). Figure 7A shows the correlation between ZC3H13 expression and tumor-infiltrating lymphocytes (TILs), and the lymphocytes with the greatest correlations included CD56dim (Spearman: ρ = -0.417, p < 2.2e-16), Tgd (Spearman: ρ = -0.286, p = 2.18e-08), eosinophils (Spearman: ρ = 0.272, p = 1.03e-07), and Act-CD8 (Spearman: ρ = -0.269, p = 1.52e-07) (Figure 7B). Immunomodulators can be further classified into immunoinhibitors, immunostimulators, and major histocompatibility complex (MHC) molecules. Figure S6 shows correlations between ZC3H13 expression levels and immunoinhibitors. The immunoinhibitors with the greatest correlations included KDR (Spearman: ρ = 0.468, p < 2.2e-16), PVRL2 (Spearman: ρ = -0.261, p = 3.42e-07), LAG3 (Spearman: ρ = -0.261, p = 3.38e-07), and LGALS9 (Spearman: ρ = -0.255, p = 6.8e-07). Figure S7 shows correlations between ZC3H13 expression and immunostimulators, and the immunostimulators with the greatest correlations included IL6R (Spearman: ρ = 0.424, p < 2.2e-16), TNFRSF18 (Spearman: ρ = -0.407, p < 2.2e-16), TNFRSF4 (Spearman: ρ = -0.369, p = 2.45e-13), and TNFRSF14 (Spearman: ρ = -0.287, p = 1.93e-08). Figure S8 shows correlations between ZC3H13 expression and MHC molecules, and the MHC molecules with the greatest correlations included TAPBP (Spearman: ρ = -0.309, p = 1.33e-09), HLA-DMA (Spearman: ρ = -0.226, p = 1.08e-05), HLA-DOB (Spearman: ρ = -0.214, p = 3.1e-05), and TAP1 (Spearman: ρ = -0.211, p = 4.04e-05). Therefore, ZC3H13 may be involved in regulating the above immune molecules.

## Discussion

It is well documented that the genetic and epigenetic alterations induced by m6A RNA methylation regulators modulate the related phenotypes. Aberrantly expressed m6A RNA methylation regulators have been correlated with various malignant behaviors in multiple cancer types. For HCC, previous studies indicated that some m6A RNA methylation regulators, such as KIAA1429, WTAP, and FTO, were overexpressed in tissues and cell lines. However, despite recent advances in the field, the roles of m6A modification in liver cancer remain largely unknown.

In this study, we examined 12 widely reported m6A RNA methylation regulators in TCGA LIHC datasets. Here, we compared mRNA expression levels of m6A-related genes between HCC and normal liver tissues using the Oncomine and GEPIA databases and analyzed the prognostic value of each m6A family member in liver cancer using the Kaplan-Meier plotter database. Because of the importance of the m6A family members in HCC, genetic alteration analysis of the m6A family members was performed. The above-mentioned results revealed that ZC3H13 might be of significance in HCC and might serve as a biomarker in diagnosis and prognosis. In addition, its expression level was correlated with tumor grade. Thus, we selected ZC3H13 for further analysis.

ZC3H13 (zinc finger CCCH domain‐containing protein 13) is a classical CCCH zinc finger protein located on human chromosome 13q14.13 28. ZC3H13 zinc finger protein acts as a recruiter protein promoting localization of the writer complex in the nucleus. Zhu et al. indicated that ZC3H13 may be an upstream regulator of Ras-ERK signaling pathway and suppressed invasion and proliferation of colorectal cancer 29. However, Gewurz et al. reported that ZC3H13 might be a pivotal upstream factor of NF‐κB, which was responsible for its activation. Activation of NF‐κB signaling was confirmed to accelerate tumor proliferation and invasion 30, which suggested that ZC3H13 might be an oncogenic protein. Luo et al. showed that ZC3H13 might bind with K‐Ras, which is frequently mutated in various cancers, such as non‐small cell lung cancer (NSCLC) and colon carcinoma 31, 32, and is tightly associated with cancer progression. To date, the biological role of ZC3H13 is still unknown, and only a few studies have focused on its expression in HCC. In this study, ZC3H13 expression was significantly downregulated in liver tumor tissues. Survival outcome analysis suggested that significant correlations existed between ZC3H13 downregulation and poor OS and poor RFS in patients with liver cancer, suggesting its role as a tumor suppressor gene. To further uncover the roles of ZC3H13 in HCC, the current study conducted functional assays *in vitro* and *in vivo*. Upregulating ZC3H13 inhibited the aggressive behaviors of HCC cells.

To explore its biological functions and relevant pathways, gene set enrichment analysis was performed on genes co-expressed with ZC3H13 in TCGA database. In our study, the enrichment analysis showed that the biological process results enriched in the co-expressed genes included transcriptional misregulation in cancer and negative regulation of the cell cycle. The JNK MAPK pathway and signaling by TGFB family members have been found to be involved. Sustaining mitogenic signaling and evading growth suppressors in tumor cells are potentially the most fundamental characteristics of tumor cells, unlike in normal cells, which regulate cell homeostasis, especially by releasing growth-promoting signals 33. For instance, HCC arises largely as a result of uninhibited cellular proliferation resulting from a series of dysregulations in normal cell cycle regulators such as cyclin-dependent kinases (CDKs). Given the unique regenerative capacity of hepatocytes, any reprobation of cell proliferation, upregulation of CDKs, or alterations in CDK-related downstream signaling pathways and CDK inhibitors could potentiate the onset of hepatocarcinogenesis 34.

In our study, we assessed the correlation between ZC3H13 and the immune system in the TISIDB database, and the results showed that ZC3H13 had the greatest correlation with lymphocytes (such as CD56dim, Tgd, eosinophil and Act-CD8), immunoinhibitors (such as KDR, LAG3, PVRL2 and LGALS9), immunostimulators (such as TNFSF4, TNFSF18, TNFSF14, and IL6R), and MHC molecules (such as HLA-DMA, HLA-DOB, DPA1, and TAPBP). LIGHT, also known as tumor necrosis factor superfamily member 14 (TNFSF14), is one of the costimulatory molecules that can regulate T-cell activation 35. TNFSF14 in particular has shown promise as an immunomodulator that can enhance T cell and natural killer (NK)-cell proliferation, function, and anti-tumor responses 36. Therefore, it appears that TNFSF14 (LIGHT) in the tumor microenvironment may reflect or contribute to an anti-tumor immune response. In addition, cellular metabolic switches in the liver can regulate the pre-tumor function of monocytes in a specific human tumor microenvironment 37. Although the underlying mechanism of HCC-induced immunosuppression has not been determined, there is growing evidence that immunotherapy that harnesses anti-tumor immunity can be accomplished by changing the function or number of immune cells, the expression of immune receptors or ligands, and the level of cytokines.

## Conclusion

M6A family members display varying degrees of abnormal expression and play important roles in the progression of HCC. Our data revealed that ZC3H13 was expressed at significantly low levels in malignant liver tumor tissues and that low ZC3H13 expression was significantly correlated with poor prognosis in patients with HCC. Furthermore, ZC3H13 was further identified as a tumor suppressor gene for HCC by functional assays *in vitro* and *in vivo*. In addition, we showed that ZC3H13 had the greatest correlation with lymphocytes. Therefore, ZC3H13 may prove to be a novel biomarker for the early diagnosis, immunotherapy, and prognostic judgment of patients with HCC.

## Supplementary Material

Supplementary figures and table.

## Figures and Tables

**Table 1:** Datasets of m6A-related genes in hepatocellular carcinoma (Oncomine)

Table 1. Datasets of m6A-related genes in hepatocellular carcinoma (Oncomine)						
gene	tumor(cases)	normal(cases)	fold change	t-test	p-value	dataset
KIAA1429	Hepatocellular Carcinoma (35)	liver(10)	2.117	7.522	1.17E-09	Wurmbach et al.
RBM15	Hepatocellular Carcinoma (225)	liver(220)	1.324	8.681	4.53E-17	Roessler 2 et al.
WTAP	Hepatocellular Carcinoma (225)	liver(220)	1.42	8.763	2.81E-17	Roessler 2 et al.
ZC3H13	Hepatocellular Carcinoma(22)	liver(21)	-3.105	-9.418	1.54E-11	Roessler et al.
HNRNPC	Hepatocellular Carcinoma(22)	liver(21)	1.551	7.65	5.44E-09	Roessler et al.
YTHDF1	Hepatocellular Carcinoma(26)	liver(26)	1.044	4.5	3.63E-05	Guichard 2 et al.
YTHDC1	Hepatocellular Carcinoma(104)	liver(76)	1.888	10.315	5.48E-20	Chen et al.
YTHDF2	Hepatocellular Carcinoma(35)	liver(10)	1.44	3.639	2.00E-03	Wurmbach et al.
YTHDF3	Hepatocellular Carcinoma(99)	liver(86)	1.106	7.092	9.24E-11	Guichard et al.
ALKBH5	Hepatocellular Carcinoma(104)	liver(76)	1.171	2.41	9.00E-03	Chen et al.
FTO	Hepatocellular Carcinoma(26)	liver(26)	-1.056	-3.225	2.00E-03	Guichard 2 et al.

## Abbreviations

m6A: N6-methyladenosine
LIHC: liver hepatocellular carcinoma
HCC: hepatocellular carcinoma
HBV: hepatitis B virus
HCV: hepatitis C virus
TCGA: The Cancer Genome Atlas
GO: Gene Ontology
KEGG: Kyoto Encyclopedia of Genes and Genomes
ZC3H13: zinc finger CCCH-type containing 13
OS: Overall survival
RFS: Recurrence free survival
qPCR: Quantitative Real-time PCR
GSEA: Gene set enrichment analysis
RBM15: RNA-binding motif protein 15
METTL14: methyltransferase like 14
WTAP: Wilms tumor 1 associated protein
FTO: fat mass- and obesity-associated protein
ALKBH5: alkb homolog 5
HNRNPC: heterogeneous nuclear ribonucleoprotein C
