# Supplementary material for: Identification and validation of the N6-methyladenosine RNA methylation regulator ZC3H13 as a novel prognostic marker and potential target for hepatocellular carcinoma
Source: Int J Med Sci. 2022 Mar 21;19(4):618–30. doi: 10.7150/ijms.69645 (PMC9108408; doi:10.7150/ijms.69645)
Supplement: Supplementary file 1 — Supplementary figures and table. [file ijmsv19p0618s1.pdf]

Supplementary

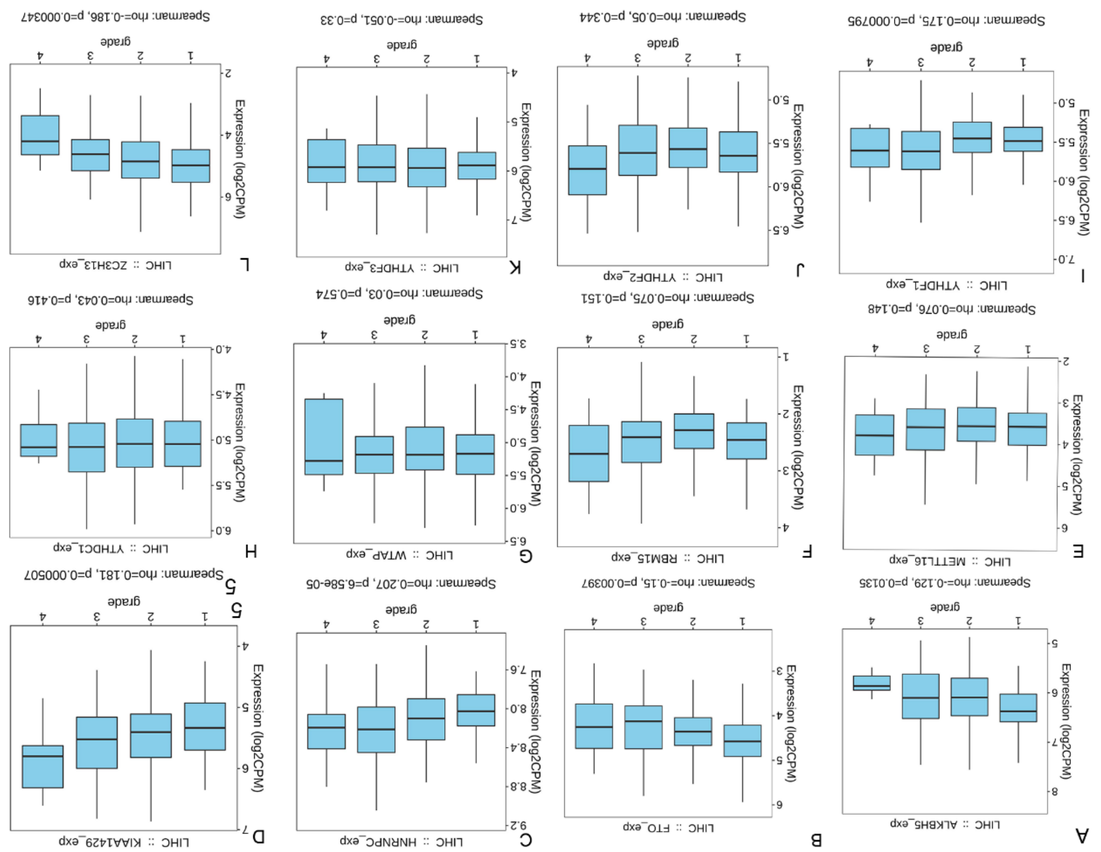

Figure S1. Correlation analysis of the m6A-related genes expression and tumor grades in LIHC(TISIDB). (A-L) Correlation between the expression of each m6A-related genes and tumor grades in hepatocellular carcinoma. Spearman's correlation analysis between biomarkers and tumor grades is based on whole tumor grades. Grade 1 is highly differentiated, grade 2 is moderately differentiated, grade 3 is poorly differentiated and grade 4 is undifferentiated.

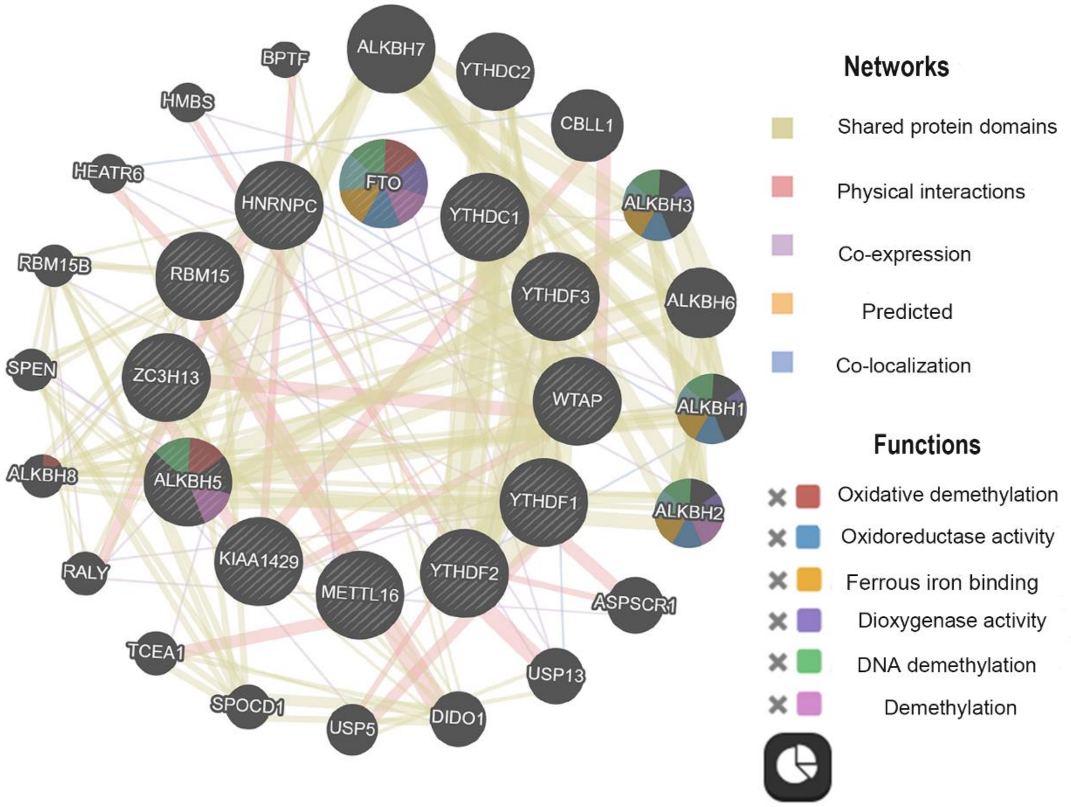

Figure S2. Gene–gene interaction network among m6A family members (GeneMANIA). Each node represents a gene. The node size represents the strength of interactions. The inter-node connection lines represent the types of gene–gene interactions, and the line color represents the types of interactions.

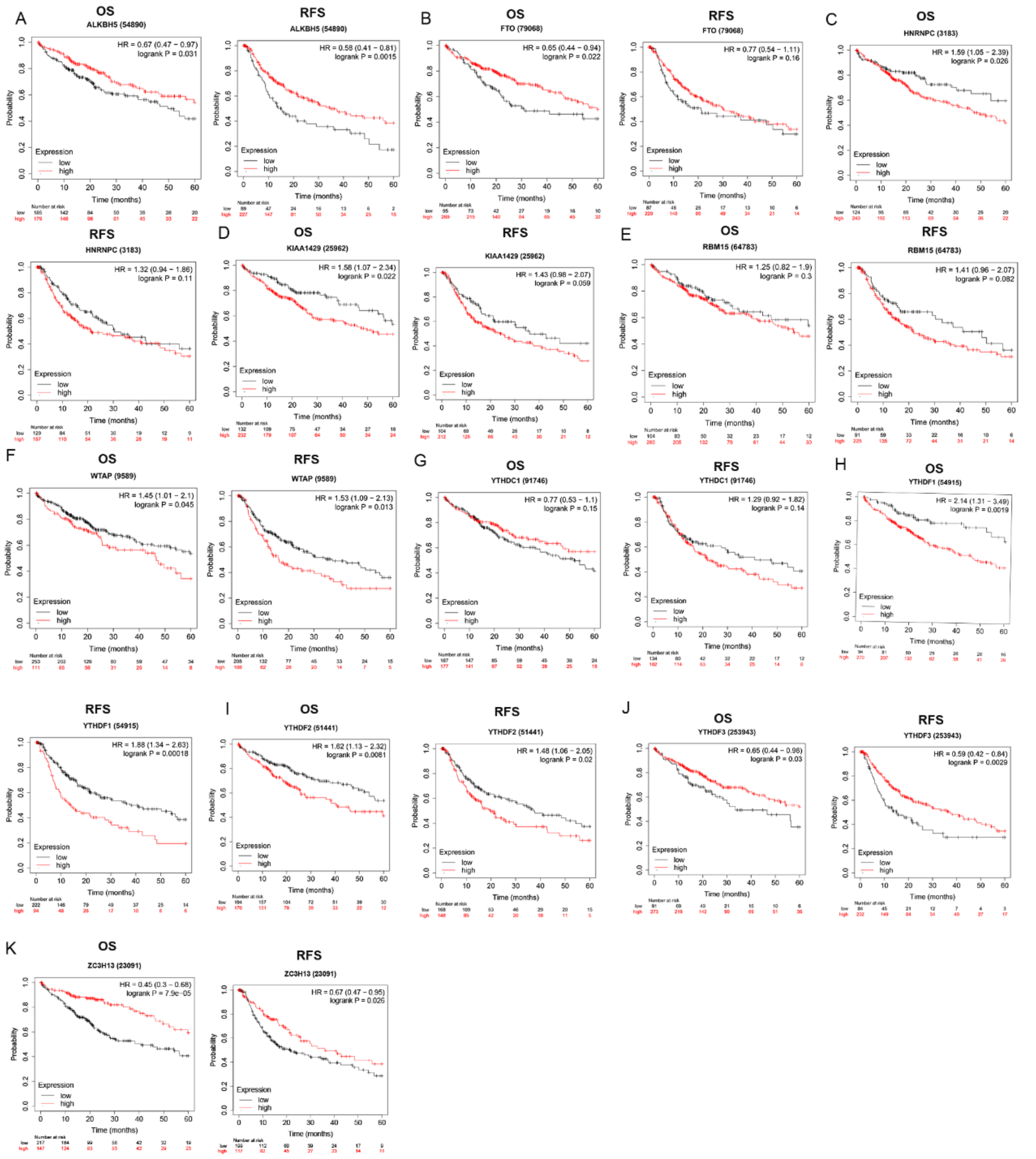

Figure S3. The prognostic value of m6A-related genes in LIHC (KM plotter). (A) LIHC patients with high ALKBH5 mRNA level had a better OS, and PFS. (B) LIHC patients with high FTO mRNA level had a better OS. (C-D) LIHC patients with low HNRNPC or KIAA1429 mRNA level had a better OS. (E) The mRNA levels of RBM15 had no effect on LIHC patients' prognosis (OS, and PPS). (F) LIHC patients with low WTAP mRNA level had a better OS, and PFS. (G) The mRNA levels of YTHFC1 had no effect on LIHC patients' prognosis (OS, and PPS). (H-I) LIHC patients with high YTHDF1 or YTHDF2 mRNA level had a worse OS and RFS. (J-K) LIHC patients with high

YTHDF3 and ZC3H13 mRNA level had a better OS and RFS. All the analyses were performed with Kaplan-Meier analysis. HR, Hazard Ratio; OS, Overall survival; RFS, Recurrence free survival.

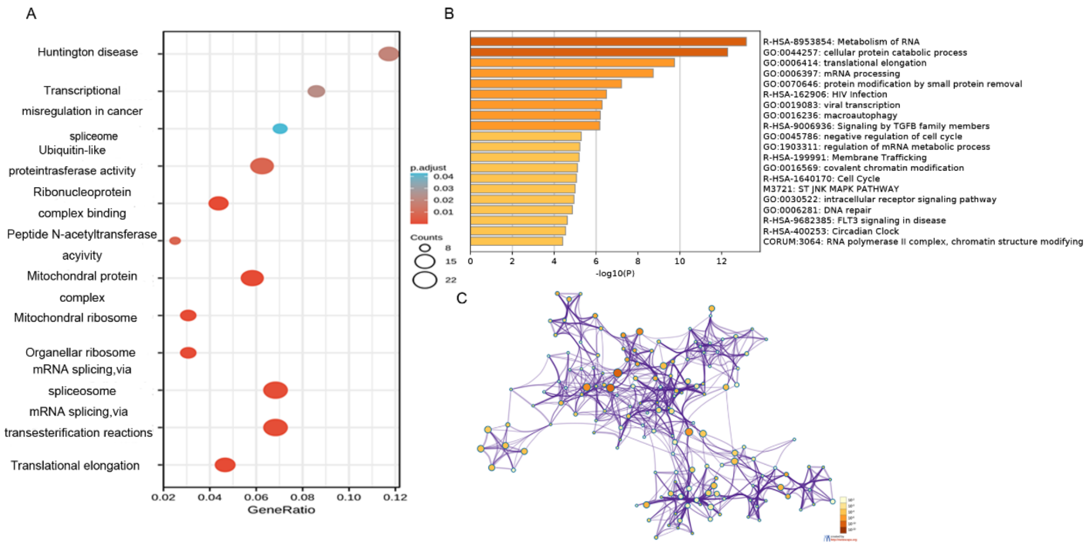

Figure S4. Functional enrichment analysis of genes co-expressed with ZC3H13 (Metascape). (A) ZC3H13 and co-expressed genes ontology (GO) enriched terms, colored by P-values(xiantao academic). (B) ZC3H13 and co-expressed genes ontology (GO) enriched terms, colored by P-values(Metascape). (C) Network of GO enriched terms colored by P-value, where terms containing more genes tend to have a more significant P-value(Metascape).

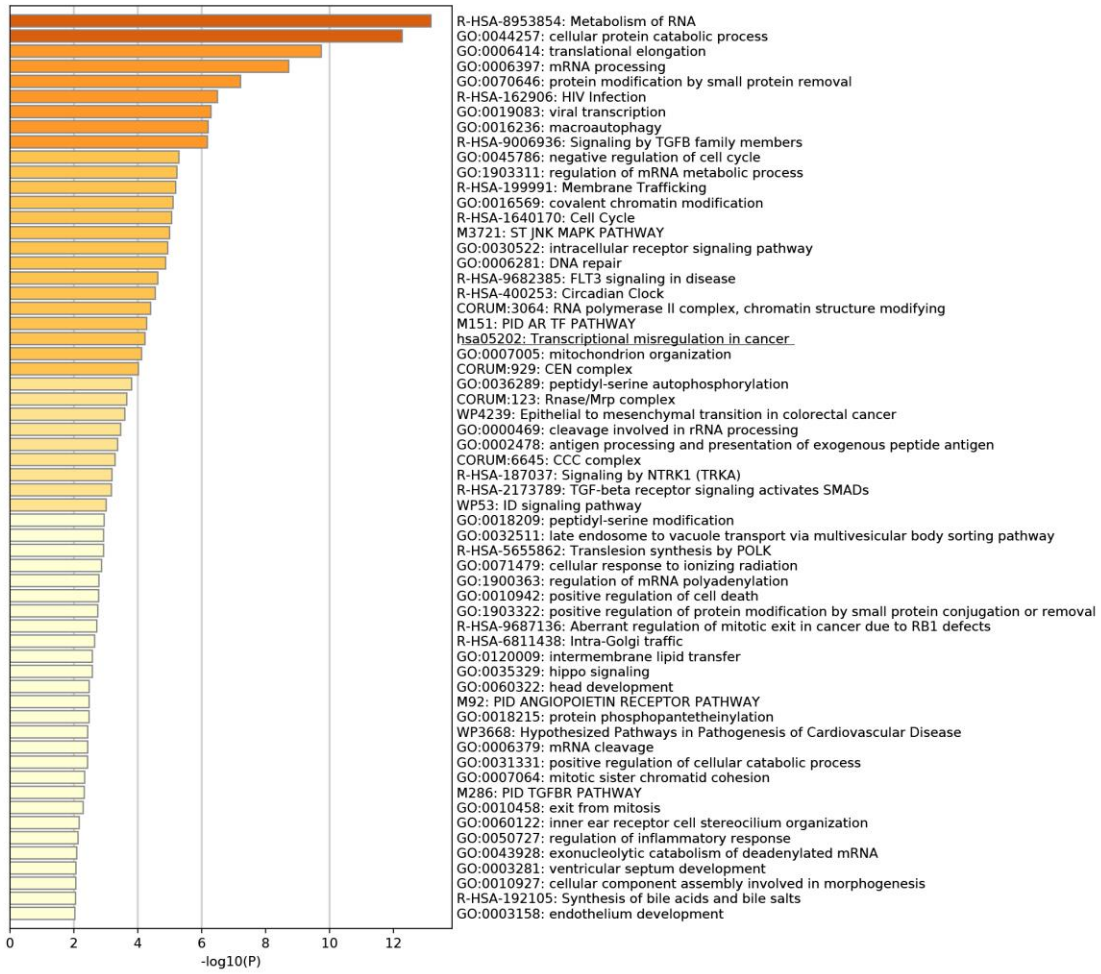

Figure S5. Enrichment-heatmap HeatmapSelected GO Top100(Metascape).

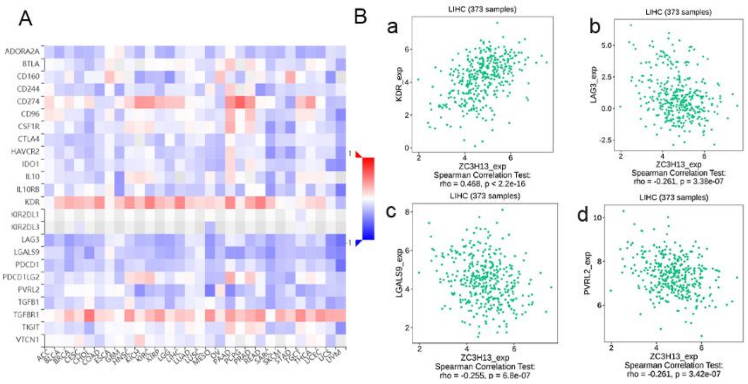

Figure S6. Spearman's correlation of ZC3H13 with immunoinhibitors (TISIDB) in LIHC.

(A) Relations between the abundances of immunoinhibitors and ZC3H13 expression.

(B) Top 4 immunoinhibitors displaying the greatest Spearman's correlation with ZC3H13

expression.

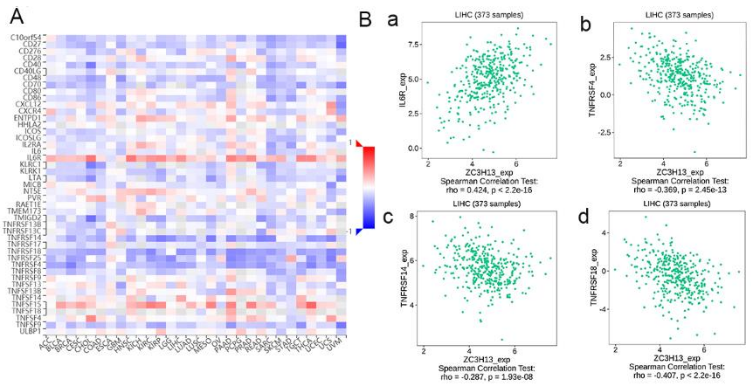

Figure S7. Spearman's correlation of ZC3H13 with immunostimulators (TISIDB) in LIHC.

(A) Relations between abundances of immunostimulators and ZC3H13 expression.

(B) Top 4 immunostimulators displaying the greatest Spearman's correlation with ZC3H13 expression.

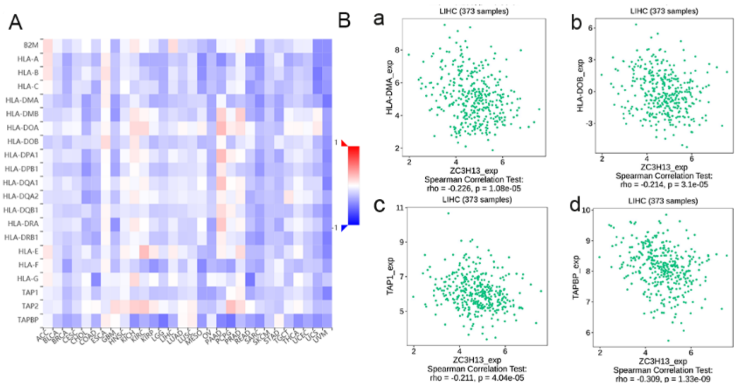

Figure S8. Spearman's correlation of ZC3H13 with MHC (TISIDB) in LIHC.

(A) Relations between abundance of MHC molecules and ZC3H13 expression.

(B) Top 4 MHC molecules displaying the greatest Spearman's correlation with ZC3H13 expression. MHC major histocompatibility complex.

Table S1 The genes co-expressed with ZC3H13 having an average Spearman's correlation coefficient of 0.5

Correlated Gene	Cytoband	Spearman's Correlation	p-Value	q-Value
FNDC3A	13q14.2	0.758692729	2.26E-66	4.53E-62
AKAP11	13q14.11	0.701788759	6.67E-53	6.69E-49
VPS36	13q14.3	0.691218675	9.53E-51	6.38E-47
VWA8	13q14.11	0.683057768	3.81E-49	1.91E-45
UTP14C	13q14.3	0.671306585	6.27E-47	2.52E-43
COG3	13q14.13	0.66482934	9.46E-46	3.17E-42
CHD9	16q12.2	0.652409875	1.43E-43	4.10E-40
KBTBD7	13q14.11	0.652053097	1.64E-43	4.13E-40

LRCH1	13q14.13-q14.2	0.65056505	2.95E-43	6.20E-40
SUCLA2	13q14.2	0.650450016	3.09E-43	6.20E-40
LATS2	13q12.11	0.638276565	3.26E-41	5.95E-38
CARF	2q33.2	0.637899286	3.76E-41	6.29E-38
CREBRF	5q35.1	0.628355686	1.25E-39	1.93E-36
SPATA13	13q12.12	0.627452209	1.73E-39	2.48E-36
KIAA1109	4q27	0.625867066	3.05E-39	4.09E-36
ELF1	13q14.11	0.624049576	5.84E-39	7.16E-36
COG6	13q14.11	0.623942515	6.06E-39	7.16E-36
GPALPP1	13q14.12	0.623502023	7.09E-39	7.69E-36
RCBTB1	13q14.2	0.623427751	7.28E-39	7.69E-36
SAC3D1	11q13.1	-0.623083741	8.22E-39	8.26E-36
USP53	4q26	0.619865905	2.55E-38	2.44E-35
ERN1	17q23.3	0.618004281	4.88E-38	4.46E-35
ERCC6L2	9q22.32	0.615518508	1.15E-37	1.01E-34
MYCBP2	13q22.3	0.613282452	2.49E-37	2.08E-34
OTUD4	4q31.21	0.612575445	3.16E-37	2.54E-34
RB1	13q14.2	0.605873299	3.02E-36	2.34E-33
LBHD1	11q12.3	-0.605306056	3.65E-36	2.72E-33
STARD13	13q13.1-q13.2	0.605140338	3.86E-36	2.77E-33
UFM1	13q13.3	0.604624675	4.58E-36	3.17E-33
NUDT1	7p22.3	-0.602011668	1.08E-35	7.26E-33
CCDC167	6p21.2	-0.601829392	1.15E-35	7.46E-33
ROMO1	20q11.22	-0.6012471	1.39E-35	8.74E-33
SNRPC	6p21.31	-0.599872949	2.18E-35	1.33E-32
PROX1	1q32.3	0.599132343	2.78E-35	1.64E-32
ABHD18	4q28.2	0.597197256	5.20E-35	2.98E-32
KAT6B	10q22.2	0.596212344	7.14E-35	3.98E-32
CCDC186	10q25.3	0.594989104	1.06E-34	5.74E-32
MEA1	6p21.1	-0.594754479	1.14E-34	6.02E-32
HIPK3	11p13	0.593777824	1.56E-34	8.02E-32
TTBK2	15q15.2	0.593616562	1.64E-34	8.23E-32
MRPL14	6p21.1	-0.592810566	2.12E-34	1.00E-31
REV3L	6q21	0.592777251	2.14E-34	1.00E-31
MPHOSPH8	13q12.11	0.592777009	2.14E-34	1.00E-31
BOD1L1	4p15.33	0.592098718	2.66E-34	1.21E-31
RBM43	2q23.3	0.592032089	2.71E-34	1.21E-31
FBXL3	13q22.3	0.589986523	5.18E-34	2.26E-31
DRAP1	11q13.1	-0.588346753	8.66E-34	3.70E-31
KLHL28	14q21.2	0.58758902	1.10E-33	4.59E-31
REST	4q12	0.587346138	1.18E-33	4.85E-31
CCNT2	2q21.3	0.58595718	1.82E-33	7.33E-31
FOXO1	13q14.11	0.585473124	2.12E-33	8.35E-31
HERC3	4q22.1	0.583927275	3.42E-33	1.32E-30

PSMG3	7p22.3	-0.581745604	6.68E-33	2.53E-30
BAZ2B	2q24.2	0.581369179	7.50E-33	2.79E-30
GEMIN7	19q13.32	-0.581021513	8.33E-33	3.04E-30
USP12	13q12.13	0.580718165	9.14E-33	3.28E-30
RBL2	16q12.2	0.580552731	9.62E-33	3.39E-30
TRAPPC8	18q12.1	0.580476421	9.84E-33	3.41E-30
DTYMK	2q37.3	-0.579670141	1.26E-32	4.28E-30
MTMR6	13q12.13	0.577125143	2.72E-32	9.09E-30
MYO9A	15q23	0.575781459	4.07E-32	1.34E-29
DYNLRB1	20q11.22	-0.575254407	4.76E-32	1.54E-29
POP7	7q22.1	-0.574534017	5.90E-32	1.88E-29
LSM2	6p21.33	-0.573390505	8.30E-32	2.60E-29
SECISBP2L	15q21.1	0.572662712	1.03E-31	3.14E-29
UQCC3	11q12.3	-0.572660719	1.03E-31	3.14E-29
ARHGEF12	11q23.3	0.57189591	1.29E-31	3.88E-29
NABP2	12q13.3	-0.570734215	1.82E-31	5.38E-29
DNAJB14	4q23	0.570136792	2.17E-31	6.32E-29
ZNF791	19p13.13	0.569973351	2.28E-31	6.54E-29
POLR2H	3q27.1	-0.569130524	2.92E-31	8.25E-29
FAM214A	15q21.2-q21.3	0.566786551	5.78E-31	1.61E-28
LMO7	13q22.2	0.566576129	6.15E-31	1.69E-28
PDS5B	13q13.1	0.566317871	6.63E-31	1.80E-28
IL6ST	5q11.2	0.566194009	6.87E-31	1.84E-28
INTS6	13q14.3	0.564403285	1.15E-30	3.02E-28
ZEB1	10p11.22	0.564394173	1.16E-30	3.02E-28
SNRPD2	19q13.32	-0.563957384	1.31E-30	3.38E-28
WWC2	4q35.1	0.563544512	1.48E-30	3.75E-28
EPC2	2q23.1	0.562994863	1.73E-30	4.34E-28
PQBP1	Xp11.23	-0.56276091	1.85E-30	4.58E-28
TRPM7	15q21.2	0.562648723	1.91E-30	4.68E-28
RRP1	21q22.3	-0.562136762	2.21E-30	5.35E-28
TSSC4	11p15.5	-0.561660109	2.53E-30	6.06E-28
PSMD13	11p15.5	-0.561588924	2.59E-30	6.11E-28
AKAP2	9q31.3	0.561538855	2.62E-30	6.12E-28
AP1G1	16q22.2	0.561294788	2.81E-30	6.49E-28
TOGARAM1	14q21.2	0.560881632	3.16E-30	7.22E-28
SCNM1	1q21.3	-0.560779126	3.26E-30	7.35E-28
USPL1	13q12.3	0.560370241	3.66E-30	8.17E-28
SYNE2	14q23.2	0.559907824	4.17E-30	9.21E-28
DAAM1	14q23.1	0.558308445	6.56E-30	1.43E-27
REL	2p16.1	0.558083786	6.99E-30	1.51E-27
SNF8	17q21.32	-0.557714194	7.76E-30	1.66E-27
FRYL	4p11	0.557613294	7.99E-30	1.69E-27
BIRC6	2p22.3	0.557536802	8.16E-30	1.71E-27

RRP36	6p21.1	-0.557472451	8.31E-30	1.72E-27
RFXANK	19p13.11	-0.556730136	1.02E-29	2.10E-27
LAMTOR4	7q22.1	-0.555987536	1.26E-29	2.56E-27
RPP21	6p22.1	-0.555807012	1.33E-29	2.66E-27
ARL6IP4	12q24.31	-0.555646704	1.39E-29	2.76E-27
ZKSCAN1	7q22.1	0.555524835	1.44E-29	2.83E-27
UBE2S	19q13.42	-0.555289926	1.53E-29	2.99E-27
GTF2A1	14q31.1	0.555090221	1.62E-29	3.13E-27
TET2	4q24	0.55440638	1.96E-29	3.75E-27
METTL14	4q26	0.553570568	2.48E-29	4.69E-27
SERINC1	6q22.31	0.553454782	2.56E-29	4.80E-27
DHX33	17p13.2	0.552784506	3.08E-29	5.70E-27
MAP3K2	2q14.3	0.5527697	3.09E-29	5.70E-27
UBXN4	2q21.3	0.552379647	3.44E-29	6.29E-27
HEATR5B	2p22.2	0.552048456	3.77E-29	6.80E-27
CCNT1	12q13.11-q13.12	0.552029586	3.79E-29	6.80E-27
CPSF4	7q22.1	-0.551830346	4.01E-29	7.13E-27
RPS21	20q13.33	-0.550630455	5.58E-29	9.83E-27
SPG11	15q21.1	0.549959325	6.71E-29	1.17E-26
NAA10	Xq28	-0.549753174	7.10E-29	1.23E-26
COMMD4	15q24.2	-0.549483526	7.65E-29	1.31E-26
ZDHHC20	13q12.11	0.549238601	8.18E-29	1.39E-26
UBR3	2q31.1	0.548271107	1.07E-28	1.80E-26
SLC25A39	17q21.31	-0.547226969	1.42E-28	2.37E-26
TSTD2	9q22.33	0.546875885	1.56E-28	2.59E-26
DIS3	13q21.33	0.546398947	1.77E-28	2.92E-26
JMJD1C	10q21.3	0.546365063	1.79E-28	2.92E-26
PIKFYVE	2q34	0.546262557	1.84E-28	2.98E-26
LIG4	13q33.3	0.546192796	1.88E-28	3.01E-26
BCL7C	16p11.2	-0.545681974	2.15E-28	3.43E-26
GBAP1	1q22	-0.545285048	2.40E-28	3.79E-26
MYSM1	1p32.1	0.545196494	2.46E-28	3.85E-26
BRMS1	11q13.2	-0.54510253	2.52E-28	3.92E-26
POP5	12q24.31	-0.544785616	2.74E-28	4.24E-26
DPP8	15q22.31	0.544176843	3.23E-28	4.96E-26
MINDY2	15q21.3-q22.1	0.54414239	3.26E-28	4.97E-26
BEND3P3	10q22.3	0.54407975	3.32E-28	5.01E-26
ZNF366	5q13.2|5q13.2	0.54328585	4.11E-28	6.16E-26
MXD3	5q35.3	-0.54280629	4.67E-28	6.95E-26
FBL	19q13.2	-0.542281051	5.38E-28	7.95E-26
UBR1	15q15.2	0.542202747	5.49E-28	8.05E-26
SLTM	15q22.1	0.542038776	5.74E-28	8.31E-26
KLHDC3	6p21.1	-0.542033328	5.75E-28	8.31E-26
CIPC	14q24.3	0.541924842	5.92E-28	8.49E-26

ASXL2	2p23.3	0.541801265	6.12E-28	8.71E-26
PCDHGB7	5q31.3	0.541433326	6.75E-28	9.54E-26
PGP	16p13.3	-0.541392665	6.82E-28	9.58E-26
RAD54L2	3p21.2	0.541230648	7.12E-28	9.93E-26
LTN1	21q21.3	0.540916866	7.74E-28	1.07E-25
NELFE	6p21.33	-0.540531898	8.58E-28	1.18E-25
KPNA3	13q14.2	0.53999488	9.90E-28	1.35E-25
SHPRH	6q24.3	0.539940391	1.00E-27	1.36E-25
TRIP11	14q32.12	0.539627282	1.09E-27	1.47E-25
CLASP1	2q14.2-q14.3	0.539429958	1.15E-27	1.54E-25
MAGI1	3p14.1	0.539079729	1.26E-27	1.68E-25
UQCC2	6p21.31	-0.538330011	1.54E-27	2.03E-25
TIMM50	19q13.2	-0.53810222	1.63E-27	2.14E-25
CLOCK	4q12	0.537908597	1.72E-27	2.24E-25
POLK	5q13.3	0.53776936	1.78E-27	2.31E-25
USF3	3q13.2	0.537411443	1.96E-27	2.52E-25
ATF2	2q31.1	0.537161157	2.09E-27	2.68E-25
RNF6	13q12.13	0.537123856	2.11E-27	2.69E-25
COPS6	7q22.1	-0.536968427	2.20E-27	2.78E-25
ERI3	1p34.1	-0.536649196	2.39E-27	3.01E-25
POLR2I	19q13.12	-0.536381541	2.57E-27	3.20E-25
BCL2L12	19q13.33	-0.53599572	2.84E-27	3.52E-25
CDK8	13q12.13	0.535624857	3.13E-27	3.86E-25
NEMF	14q21.3	0.535589578	3.16E-27	3.87E-25
ANAPC11	17q25.3	-0.535512233	3.23E-27	3.93E-25
PIK3C2A	11p15.1	0.535456424	3.27E-27	3.96E-25
RAPGEF6	5q31.1	0.53519902	3.50E-27	4.21E-25
PPTC7	12q24.11	0.53514976	3.55E-27	4.24E-25
ANKRD17	4q13.3	0.535070602	3.62E-27	4.30E-25
DDI2	1p36.21	0.534987763	3.70E-27	4.35E-25
SNRPB	20p13	-0.534984611	3.70E-27	4.35E-25
TRAPPC11	4q35.1	0.534503117	4.20E-27	4.90E-25
WDFY3	4q21.23	0.53416033	4.59E-27	5.33E-25
ZNF281	1q32.1	0.533706133	5.17E-27	5.96E-25
THAP8	19q13.12	-0.533678266	5.20E-27	5.97E-25
RAPGEF2	4q32.1	0.533423102	5.56E-27	6.35E-25
ARL15	5q11.2	0.533275322	5.78E-27	6.56E-25
PLEKHM3	2q33.3	0.532310057	7.42E-27	8.37E-25
CHCHD1	10q22.2	-0.531698722	8.69E-27	9.75E-25
N4BP2	4p14	0.531608925	8.90E-27	9.93E-25
LATS1	6q25.1	0.531505669	9.14E-27	1.01E-24
WBP4	13q14.11	0.531299518	9.64E-27	1.06E-24
RUNX1T1	8q21.3	0.531170539	9.96E-27	1.09E-24
USO1	4q21.1	0.531143481	1.00E-26	1.10E-24

ACOT8	20q13.12	-0.531053219	1.03E-26	1.11E-24
EPC1	10p11.22	0.530927079	1.06E-26	1.15E-24
UHMK1	1q23.3	0.530556064	1.17E-26	1.25E-24
RSPRY1	16q13	0.530427362	1.21E-26	1.29E-24
ALG3	3q27.1	-0.53005834	1.33E-26	1.41E-24
C19ORF53	19p13.13	-0.529462666	1.55E-26	1.63E-24
CDC14C	7p12.3	0.529350689	1.59E-26	1.66E-24
NDFIP2	13q31.1	0.529348239	1.59E-26	1.66E-24
KRTCAP2	1q22	-0.52928869	1.62E-26	1.68E-24
OSBP	11q12.1	0.529249396	1.63E-26	1.69E-24
KLHDC1	14q21.3	0.528764808	1.85E-26	1.90E-24
RBM42	19q13.12	-0.528564884	1.95E-26	1.99E-24
LAGE3	Xq28	-0.52772889	2.41E-26	2.45E-24
RPLP2	11p15.5	-0.527404287	2.62E-26	2.65E-24
MRPS17	7p11.2	-0.527321997	2.67E-26	2.70E-24
SMARCA2	9p24.3	0.527016472	2.89E-26	2.90E-24
ATXN1L	16q22.2	0.526918807	2.96E-26	2.96E-24
ZKSCAN8	6p22.1	0.526825128	3.03E-26	3.01E-24
TLCD1	17q11.2	-0.526712693	3.12E-26	3.09E-24
BOLA2	16p11.2	-0.526566015	3.24E-26	3.19E-24
ITPRID2	2q31.3	0.526179055	3.57E-26	3.50E-24
POLR2A	17p13.1	0.525818575	3.91E-26	3.81E-24
TGFBRAP1	2q12.1-q12.2	0.525212028	4.56E-26	4.43E-24
NDUFB11	Xp11.3	-0.525005255	4.81E-26	4.64E-24
RAB3GAP1	2q21.3	0.524976317	4.84E-26	4.65E-24
BDP1	5q13.2	0.524923355	4.91E-26	4.69E-24
AP2S1	19q13.32	-0.52489602	4.94E-26	4.70E-24
KLHL8	4q22.1	0.524640894	5.27E-26	4.99E-24
ELF2	4q31.1	0.524337647	5.69E-26	5.34E-24
LEPROT	1p31.3	0.524333376	5.69E-26	5.34E-24
RNF111	15q22.1-q22.2	0.524263045	5.80E-26	5.41E-24
NFKBIL1	6p21.33	-0.523993967	6.20E-26	5.77E-24
CUTA	6p21.32	-0.523693567	6.69E-26	6.19E-24
SMG1	16p12.3	0.523655982	6.75E-26	6.22E-24
NDUFS6	5p15.33	-0.523357575	7.28E-26	6.68E-24
DENND4A	15q22.31	0.523101025	7.76E-26	7.09E-24
COMMD9	11p13	-0.522758769	8.46E-26	7.69E-24
UFL1	6q16.1	0.522737983	8.50E-26	7.69E-24
ZNF397	18q12.2	0.522294644	9.50E-26	8.56E-24
LARP1B	4q28.2	0.522276705	9.55E-26	8.56E-24
SCAF11	12q12	0.522210077	9.71E-26	8.66E-24
KIF27	9q21.32	0.521954276	1.03E-25	9.20E-24
PSMB3	17q12	-0.521757341	1.09E-25	9.62E-24
NEK1	4q33	0.521606399	1.13E-25	9.94E-24

NCAPH2	22q13.33	-0.521587352	1.13E-25	9.95E-24
CENPC	4q13.2	0.521518445	1.15E-25	1.01E-23
CSNK2B	6p21.33	-0.521499652	1.16E-25	1.01E-23
SNRK	3p22.1	0.52066793	1.43E-25	1.23E-23
TIMM17B	Xp11.23	-0.519935011	1.71E-25	1.48E-23
TNRC6A	16p12.1	0.519649133	1.84E-25	1.58E-23
MRPL51	12p13.31	-0.519450955	1.93E-25	1.65E-23
MRPL11	11q13.2	-0.519265874	2.02E-25	1.72E-23
SURF2	9q34.2	-0.519259895	2.02E-25	1.72E-23
TOPORS	9p21.1	0.519126352	2.09E-25	1.76E-23
8-Mar	10q11.21-q11.22	0.5190956	2.11E-25	1.76E-23
NBEAL1	2q33.2	0.519089336	2.11E-25	1.76E-23
PTTG1	5q33.3	-0.51908478	2.11E-25	1.76E-23
SEC24B	4q25	0.51861553	2.37E-25	1.97E-23
ATM	11q22.3	0.518397705	2.51E-25	2.07E-23
LACC1	13q14.11	0.518258467	2.59E-25	2.13E-23
TXNL4A	18q23	-0.517799753	2.90E-25	2.38E-23
NR2C2AP	19p13.11	-0.517550036	3.09E-25	2.52E-23
ZMYND11	10p15.3	0.517372075	3.23E-25	2.62E-23
ATP5MC2	12q13.13	-0.517241095	3.33E-25	2.70E-23
ATE1	10q26.13	0.517173327	3.39E-25	2.73E-23
C9ORF129	9q22.31	0.517156195	3.40E-25	2.73E-23
UBL3	13q12.3	0.51714229	3.42E-25	2.73E-23
DDX49	19p13.11	-0.516980843	3.55E-25	2.83E-23
KCTD18	2q33.1	0.516907132	3.62E-25	2.87E-23
MTX1	1q22	-0.516886346	3.64E-25	2.88E-23
MIIP	1p36.22	-0.516859829	3.66E-25	2.88E-23
CLTA	9p13.3	-0.516781241	3.73E-25	2.93E-23
PPP4R3B	2p16.1	0.516742126	3.77E-25	2.94E-23
CDKN2AIP	4q35.1	0.516358973	4.14E-25	3.22E-23
TRAF6	11p12	0.516323169	4.18E-25	3.24E-23
SNRPF	12q23.1	-0.516075088	4.44E-25	3.43E-23
PRELID1	5q35.3	-0.515789495	4.76E-25	3.66E-23
TMEM184C	4q31.23	0.515725998	4.84E-25	3.71E-23
NHLRC2	10q25.3	0.515636057	4.94E-25	3.77E-23
PTDSS2	11p15.5	-0.515601282	4.99E-25	3.79E-23
RPLP1	15q23	-0.515070528	5.68E-25	4.30E-23
RORA	15q22.2	0.514644559	6.30E-25	4.76E-23
RGPD3	2q12.2	0.514527816	6.48E-25	4.88E-23
KIAA2026	9p24.1	0.514456346	6.60E-25	4.94E-23
ZMYM2	13q12.11	0.514257883	6.92E-25	5.17E-23
DPY19L3	19q13.11	0.514093019	7.21E-25	5.36E-23
PYM1	12q13.2	-0.513723428	7.89E-25	5.82E-23
CENPM	22q13.2	-0.513723143	7.89E-25	5.82E-23

MEF2A	15q26.3	0.513583906	8.16E-25	6.00E-23
COL4A3BP	5q13.3	0.51326215	8.82E-25	6.47E-23
COMMD7	20q11.21	-0.51306625	9.25E-25	6.76E-23
NEDD8	14q12	-0.512659964	1.02E-24	7.43E-23
MAPK8	10q11.22	0.51260629	1.03E-24	7.49E-23
KIAA0754	1p34.3	0.512596715	1.04E-24	7.49E-23
SOS2	14q21.3	0.512573082	1.04E-24	7.51E-23
FAM122A	9q21.11	0.512195803	1.14E-24	8.19E-23
B3GAT3	11q12.3	-0.512121486	1.16E-24	8.31E-23
LAMTOR1	11q13.4	-0.512017556	1.19E-24	8.50E-23
PARD3B	2q33.3	0.51184504	1.24E-24	8.83E-23
DOCK1	10q26.2	0.511793751	1.26E-24	8.91E-23
ETV3	1q23.1	0.511717236	1.28E-24	9.04E-23
SMARCAD1	4q22.3	0.511362123	1.40E-24	9.82E-23
TMEM160	19q13.32	-0.511169033	1.46E-24	1.02E-22
CLCN3	4q33	0.511119489	1.48E-24	1.03E-22
UBA52	19p13.11	-0.510940388	1.55E-24	1.08E-22
PCNX1	14q24.2	0.510642836	1.66E-24	1.15E-22
CARNMT1	9q21.13	0.510349912	1.78E-24	1.23E-22
ATG2B	14q32.2	0.510327345	1.79E-24	1.23E-22
UBE2M	19q13.43	-0.510105533	1.89E-24	1.30E-22
CLPX	15q22.31	0.510012423	1.93E-24	1.32E-22
VPS13C	15q22.2	0.509673584	2.10E-24	1.43E-22
ADAT1	16q23.1	0.509480531	2.20E-24	1.49E-22
SMAD1	4q31.21	0.509102112	2.41E-24	1.63E-22
BRI3	7q21.3	-0.508996474	2.47E-24	1.66E-22
PCDHGB2	5q31.3	0.508660828	2.68E-24	1.80E-22
TGFBR2	3p24.1	0.50843867	2.82E-24	1.89E-22
PDCD5	19q13.11	-0.508412759	2.84E-24	1.89E-22
MRPL52	14q11.2	-0.508377167	2.86E-24	1.90E-22
HIVEP1	6p24.1	0.508354103	2.88E-24	1.91E-22
ABHD12	20p11.21	-0.508271528	2.94E-24	1.94E-22
MZT2B	2q21.1	-0.507909625	3.20E-24	2.11E-22
MRPL53	2p13.1	-0.50773508	3.34E-24	2.19E-22
CDKN2AIPNL	5q31.1	-0.507245613	3.75E-24	2.45E-22
IL1R1	2q11.2-q12.1	0.507136558	3.85E-24	2.51E-22
PAFAH1B2	11q23.3	0.507037469	3.94E-24	2.56E-22
NHP2	5q35.3	-0.506954325	4.02E-24	2.61E-22
ZNF280D	15q21.3	0.506736785	4.24E-24	2.73E-22
EXOC6B	2p13.2	0.506590465	4.38E-24	2.82E-22
NR2C2	3p25.1	0.506547718	4.43E-24	2.84E-22
MRPL55	1q42.13	-0.506218844	4.79E-24	3.06E-22
ACER2	9p22.1	0.506085662	4.94E-24	3.15E-22
PRKG1	10q11.23-q21.1	0.506058314	4.98E-24	3.16E-22

ELK3	12q23.1	0.505782054	5.31E-24	3.37E-22
KLHL20	1q25.1	0.505564514	5.59E-24	3.53E-22
GGCT	7p14.3	-0.505475106	5.71E-24	3.60E-22
HRAS	11p15.5	-0.505457061	5.74E-24	3.60E-22
TANK	2q24.2	0.505433249	5.77E-24	3.61E-22
RPL27A	11p15.4	-0.505368613	5.86E-24	3.65E-22
WDR11	10q26.12	0.505015252	6.37E-24	3.96E-22
ZFPL1	11q13.1	-0.50499048	6.41E-24	3.97E-22
RSF1	11q14.1	0.504892529	6.56E-24	4.05E-22
TMF1	3p14.1	0.504593838	7.04E-24	4.34E-22
FRMD3	9q21.32	0.504549018	7.11E-24	4.37E-22
FAT4	4q28.1	0.504361919	7.43E-24	4.55E-22
MRPS24	7p13	-0.504309384	7.53E-24	4.59E-22
TOR1AIP2	1q25.2	0.504225386	7.68E-24	4.67E-22
LNPK	2q31.1	0.504185522	7.75E-24	4.70E-22
TCF12	15q21.3	0.504135693	7.84E-24	4.74E-22
PSMB4	1q21.3	-0.504114053	7.88E-24	4.75E-22
LNX2	13q12.2	0.504104656	7.90E-24	4.75E-22
LSM4	19p13.11	-0.504081877	7.94E-24	4.76E-22
YTHDC1	4q13.2	0.503934276	8.22E-24	4.91E-22
GOLIM4	3q26.2	0.503516955	9.07E-24	5.40E-22
SPTBN1	2p16.2	0.503508413	9.09E-24	5.40E-22
TRUB1	10q25.3	0.503298845	9.55E-24	5.66E-22
ANKRD50	4q28.1	0.502803399	1.07E-23	6.34E-22
SNRPA	19q13.2	-0.502619457	1.12E-23	6.60E-22
ANKRD36BP1	1q24.2	0.502370046	1.19E-23	6.97E-22
USP38	4q31.21	0.502193488	1.24E-23	7.25E-22
COPRS	17q11.2	-0.502019512	1.29E-23	7.53E-22
LCOR	10q24.1	0.501798839	1.36E-23	7.90E-22
SACM1L	3p21.31	0.501666044	1.40E-23	8.13E-22
PRDX5	11q13.1	-0.501594966	1.42E-23	8.24E-22
RNF181	2p11.2	-0.501556242	1.44E-23	8.29E-22
HECTD1	14q12	0.501199463	1.56E-23	8.99E-22
TAF10	11p15.4	-0.501095854	1.60E-23	9.18E-22
DNAJC27	2p23.3	0.500866888	1.69E-23	9.66E-22
KLHL11	17q21.2	0.500484022	1.85E-23	1.05E-21
NCOA2	8q13.3	0.500424404	1.87E-23	1.06E-21
MRPL23	11p15.5	-0.500401055	1.88E-23	1.07E-21
ZBTB38	3q23	0.500138241	2.00E-23	1.13E-21
CELF1	11p11.2	0.500108236	2.01E-23	1.14E-21
